# Sketches of American Physicians, Nathaniel Chapman, M. D.

**Published:** 1842-05-21

**Authors:** 


### *Sketches of American Physicians.*

### Nathaniel Chapman, M. D.

Professor Chapman is the Sir Henry Halford of the United States. He is not more distinguished for professional attainments than for courtliness and vivacity of manner, wit, knowledge of the world, and literary taste. His private character forms a marked contrast with that of his late friend and cotemporary, Physick, with whom he so long shared the first rank in the profession of Philadelphia. Physick, who shunned general society, and was little known except in professional intercourse, had a reserved stateliness of manner from which he never unbent. Engrossed by his patients and profession, he seldom entered into the every-day topics of life, and is remembered only as the skilful surgeon and successful operator. Chapman's temperament was cast in a different mould. Eminently social in disposition, with a gaiety of spirit that has not flagged with years—a wit—a punster—delightful as a companion, and enjoying company, he has, for a long period, occupied a position, we may say unrivalled, in the society of this city. To these brilliant qualities he unites the kindliest feelings. His wit is without malice, and he is frank, open-hearted, and open-handed. It is not, then, surprising that he is individually as popular as he is professionally eminent.

Dr. Chapman was born in Fairfax county, Virginia, on the 28th of May, 1780, and has therefore nearly completed his sixty-second year. His paternal ancestor came to Virginia with the first colony, was a captain of cavalry in the British army, and, according to an authentic tradition in the family, was the youngest son of a cousin german of Sir Walter Raleigh. The family settled on the river Pomonkey, some twenty miles from Richmond, but the branch, from which the doctor is descended, migrated about a century and a half ago to Maryland, and fixed itself on an estate on the banks of the Potomac, nearly opposite Mount Vernon, which is still in their possession. The doctor's father, however, went to Virginia, upon his marriage, where he afterwards remained.

Dr. Chapman received his early education at the Classical Academy of Alexandria, D. C., founded by General Washington, where he was six years. He subsequently spent a short time in two Colleges, though not long enough to owe either any obligation. He came to Philadelphia in the autumn of 1797, to commence the study of medicine with the late Professor Rush, of whom he became a favourite pupil. He continued three years with Rush, and in attendance upon the lectures at the University of Pennsylvania, from which he received his degree in the spring of 1800. The doctor's thesis was on hydrophobia, written, we have been told, at the request of Dr. Rush, in answer to an attack upon his favourite theory of the pathology of that disease. Dr. Chapman had, we believe, previously prepared another thesis, on the sympathetic connections of the stomach with the rest of the body, which he afterwards read before the Philadelphia Medical Society. This contained the substance of the peculiar views on fever and other diseases, as well as the modus operandi of medicines, which he has since taught. While a student, Chapman found time to become a frequent contributor to the Port Folio, a magazine of some celebrity in its day. His contributions, under the signature of Falkland, had considerable popularity.

In 1801, he went abroad, and spent four years chiefly at Edinburgh and London. He remained a year in London, the private pupil of Abernethy, and thence passed to Edinburgh. Edinburgh was then celebrated equally for her school of medicine and her literary and scientific society. Students of medicine resorted thither, as now to Paris, from all parts of the world. Nearly all our American physicians of the olden time, Morgan, Shippen, Kuhn, Rush, Wistar, and many others received their education at Edinburgh. It may be supposed that Dr. Chapman made the most of his opportunities in the distinguished circles of the modern Athens. He was enabled to see not a little of the eminent persons of those days, and enjoyed considerable intimacy with Dugald Stewart, the Earl of Buchan, and Brougham,* then a fellow student. Before his departure from Edinburgh,

* In 1809, Dr. Chapman republished here Lord (then Mr.) Brougham's Speech before the House of Commons on the British Orders in Council, with a biographical sketch of him, in which he predicted his future eminence. Lord Brougham was then quite a young man, little known in this country.

Lord Buchan gave him a public breakfast, on the birth-day of Washington, at which a number of distinguished persons were present, when he took the occasion to entrust him with an interesting relic, valuable from a double historical association. Lord Buchan had presented to General Washington a box made of the oak that sheltered Sir William Wallace after the battle of Falkirk, with a request "to pass it, in the event of his decease, to the man in his country who should appear to merit it best." General Washington, declining so invidious a designation, returned it by will to the Earl, who committed it to Chapman, to be delivered to Dr. Rush, with a view to its being ultimately placed in the cabinet of the college at Washington, to which General Washington had bequeathed a large sum.

Dr. Chapman returned to this country in 1804. He established himself in Philadelphia, where he soon afterwards married. His attractive manners and reputation for talent secured his almost immediate success in practice. He became the favourite physician of a large portion of the higher classes of Philadelphia, and has continued, for more than thirty years, to occupy this position. He was the physician and confidential friend of the Count de Survilliers, (Joseph Bonaparte) during his long residence in Philadelphia and its vicinity. From the Count he gathered a large fund of interesting anecdote of the illustrious brother of the ex-king, and the men and scenes of his eventful times, from which the doctor occasionally draws. In his day, Dr. Chapman has seen much of the prominent statesmen of the United States, and, though never entering into politics, he is familiar with the personal history and character of most of our public men. He was summoned to the death bed of General Harrison, though too late to assist in the treatment.

As a practitioner, Dr. Chapman is distinguished as much for the charm of his manner in the sick chamber, as for skill and success in prescribing. His lively conversation and ever-ready joke are often more effective than anodyne or cordial. Indeed, in cases of trifling importance, the doctor sometimes prescribes little else. In pleasant chit-chat, both patient and physician forget the object of the visit, and the doctor will depart and 'leave no sign' for pill or bolus. But, when roused by symptoms of actual severity, Dr. Chapman is almost unequalled in resources, as he is devoted in attentions. Hence, as a consulting physician, his great powers are particularly conspicuous. Rapid and clear in diagnosis, inexhaustible in therapeutics, self-relying, never discouraged, never 'giving up the ship,' he is the physician of physicians for an emergency.

Dr. Chapman is best known abroad as a writer and a lecturer. Not long after his return home, he published a work entitled "*Select Speeches, Forensic and Parliamentary*," with critical and illustrative remarks, in five 8vo. volumes, which attracted much attention. He has since, however, confined his pen to scientific topics. The year of his return, 1804, he gave a private course upon obstetrics, which proved so popular, that, in 1806, at the age of twenty-six, he was elected adjunct to the chair of Midwifery in the University, and soon afterwards to that of the Materia Medica. His colleagues of that day, Shippen, Rush, Wistar, Physick, James, are gone, and he remains the senior professor in the University, and, doubtless, the oldest lecturer on medicine in America. The course of lectures on Materia Medica is beyond the memory of the writer of this sketch. The views and arrangement adopted by the lecturer may, however, be inferred from his "*Therapeutics*," to which allusion will be made. At the death of Rush, in 1812, Chapman was transferred to the chair of Theory and Practice, which he has ever since filled.

The lectures of Professor Chapman, annually delivered to large classes, during a period of thirty years, are of course familiar to no small portion of the profession of the United States. We but reflect general opinion, in pronouncing them erudite, elaborate, and highly finished compositions, enriched with the stores of the most varied reading and of ample personal experience. The professor has, we believe, continued to retain, as the basis of his course, the original draft at first prepared, although many lectures have been rewritten, and the whole often remodelled. Keeping pace with the progress of medical science, Professor Chapman is yet slow to adopt, certainly to give currency to what are termed the novelties of the day. On a few subjects, his opinions differ from those generally received. His views of fever are of the ultra-solidist school, and of course at variance with the prevailing doctrines. It is foreign to our purpose, however, to canvass these points critically. Dr. Chapman's delivery of his lectures is animated and emphatic. His voice is clear, not of great volume, but so highly pitched as to seem loud. A slight nasal intonation gives it a peculiarity, not unpleasant when the ear has become familiarized to it.

In addition to his courses at the University, Dr. Chapman for a long period gave clinical lectures in the hospital of the Philadelphia Alms House. He has, moreover, for upwards of twenty years delivered a summer course of lectures in the Medical Institute. This institution was founded by Dr. Chapman, although, as we learn, he has never participated in the fees, or exercised any control over the appointments to the chairs. In days of yore, the doctor was a leading debater at the Philadelphia Medical Society, when the floor of that society was a field, in which the ablest members of the profession met in earnest and often vehement discussion. Dr. Chapman has several times filled the honourable post of President of the Society. He is now the Senior Vice-President of the American Philosophical Society, and has, we believe, been chosen corresponding member of most of the learned societies of Europe.

Dr. Chapman's principal work is his "*Therapeutics*," published in 1817. It has gone through seven editions, one surreptitious; but the doctor has since refused to have it reprinted, until he finds time to bestow on it a thorough revision. The "*Therapeutics*" has enjoyed a long popularity. It is written in a very attractive style, and, as is well known, is thoroughly impregnated with most of the peculiar and original views of the author. It is perhaps hardly necessary to observe, that some of these are not in accordance with the opinions of a large portion of his professional brethren—as, for instance, the theory of the modus operandi of medicines.

In 1820, Dr. Chapman commenced the publication of the "Philadelphia Journal of the Medical and Physical Sciences," which he continued to edit for many years. The journal was undertaken with liberal views—the doctor never receiving a salary for his services. He has since been an occasional contributor to different periodicals. A large number of his lectures have been published in the previous volumes of this journal—elegantly written and standard monographs on a variety of subjects.

We feel that this sketch does very imperfect justice to one of the brightest ornaments of the profession. It has, however, the merit of being executed in a spirit of entire candour.

